# Geochemistry of Totogan Formation as potential source rock and its relationship with oil seepage manifestations in the Banjarnegara areas, Central Java, Indonesia

**DOI:** 10.1016/j.heliyon.2024.e40010

**Published:** 2024-11-02

**Authors:** Praptisih, Yoga Andriana Sendjaja, Vijaya Isnaniawardhani, Anggoro Tri Mursito, Taufik Ramli, Muhammad Maruf Mukti, Rakhmat Fakhruddin

**Affiliations:** a Faculty of Geological Engineering, Padjajaran University, Jl. Raya Bandung Sumedang Km 21 Jatinangor, Sumedang 45363, Indonesia; b Research Center for Geological Resources, National Research and Innovation Agency (BRIN), KST Samaun Samadikun, Jl. Cisitu Sangkuriang, Bandung 40135, Indonesia; c Research Center for Mining Technology, National Research and Innovation Agency (BRIN), KST Iskandar Zulkarnain, Jl. Sutami KM 15, Lampung, 35361, Indonesia

**Keywords:** Potential source rock, Oil seepage, Biomarker, Totogan formation, North serayu basin

## Abstract

The Central Java area, especially the North Serayu Basin, has the potential to accumulate hydrocarbons, as evidenced by the many manifestations of oil and gas seepage on the surface. This research in the Banjarnegara area, part of the North Serayu Basin, aims to determine the geochemistry characteristics of potential source rock of the Totogan Formation and existing oil seepage, as well as their genetic relation. Fifteen surface rock samples were analysed for the total organic carbon (TOC) content and Rock-Eval pyrolysis. Then, several samples were selected to examine the biomarker composition using GC. and GC-MS analysis. Meanwhile, the biomarker data of oil seepage data was obtained from previously published data. The identified terpanes and steranes biomarkers of potential source rock and oil seepage samples were performed to determine the relationship of oil seepage to its potential source rock. The research reveals that the Totogan Formation was deposited under suboxic to oxic conditions that can be distinguished into the estuary or bay deposit composed mainly of higher plant organic matter and the open marine deposit with input mainly from algae organic matter. Another important finding is a positive oil-to-source rock correlation between existing oil seepage and the Totogan Formation based on biomarker data. Thus, the findings of this research suggest that the Totogan Formation is the source rock of the existing oil seepage in this research area.

## 1 Introduction

Investigation of geochemical characteristics of potential hydrocarbon source rocks, e.g., quantity, type, and maturity, that are of critical importance to our understanding of source rock deposition and maturation, petroleum migration, and entrapment [1,2]. These data are essential for hydrocarbon prospect identification and evaluation within the basin. In Java Island, Indonesia, behind the present-day volcanic arc, an east-west-trending Miocene deep water basin stretches along the island's axis known as Bogor-North Serayu-Kendeng Trough [[3], [4], [5]]. In the central part of this trough, oil seeps are found in several areas of the North Serayu basin, such as in Karangkobar, Bawang, Subah, Klantung, Sojomerto, Kaliwaru, and west of Mount Ungaran [3,4]. The abundance of oil seepages is the surface manifestation of the leaking active petroleum system in the North Serayu Basin. However, its source and genetics are still unclear [6].

Muchsin et al. [7] suggest that the primary potential source rock in the Central Java area is the Eocene Karangsambung Formation and the Early Miocene Pemali Formation. Subsequently, Subroto et al. [8] conducted biomarker analysis of several potential source rocks (i.e., Halang Formation, Rambatan Formation, Pemali Formation, and Karangsambung Formation) and oil seepages. Even though only the Miocene-Pliocene Halang Formation positively correlates with oil seepage, Subroto et al. [8] concluded that the Eocene Wungkal Formation, which has similar biomarker characteristics to Halang Formation, is most likely the source rock candidate for those oil seepages due to the maturity issue of the Halang Formation [9]. However, none of the previous works, i.e., Hadimuljono & Yensusnimar [10]; Jatmiko & Praptisih [11]; Praptisih [12]; Praptisih et al. [13]; Satyana [14]; and Sutadiwiria et al. [15], discussed the potential of the Totogan Formation as a potential source rock and its geochemical characteristics in detail.

Based on the results of previous works, including by Kamtono et al. [16] in the Banyumas and Banjarnegara areas, indicate the presence of oil seeps in the Lawen area (LW-05A) [12,13]. Praptisih [12] suggested that this oil seepage is derived from the Totogan Formation, but this was postulated without any biomarker data. This paper aims to reveal the geochemical characteristics of the Totogan Formation as a promising source rock by utilising new biomarker data and discussing its relationship with the oil seepages in the North Serayu Basin.

## 2 Geological setting

**Fig. 1:**
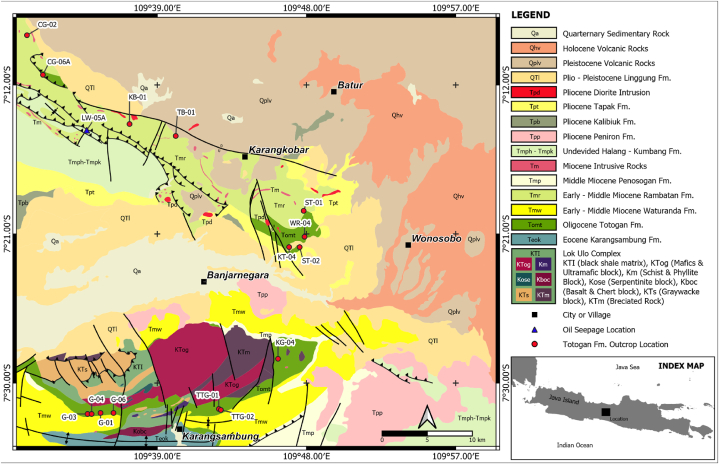
Sampling locations (source rock and oil seepage) and Geological map of Banjarnegara and its surrounding areas (modified from Asikin et al. [19] and Condon et al. [20]).

The North Serayu Basin is geographically delineated from the Banyumas Basin by the Karangbolong High to the west and from the Kebumen Basin by the Lok Ulo Plateau to the south. The Tegal and Pemalang Highs border the northern part of the North Serayu Basin. The eastern part of the North Serayu Basin is bounded by the Sumbing-Sindoro Lineament and in the southeast by the Kulon Progo High [17]. Asikin [18] studied the tectonostratigraphic and tectonic evolution of the Banjarnegara area and identified the oldest rock in this area as the Pre-Tertiary Luk Ulo melange complex. The Tertiary sedimentary rocks, consisting of olistostrome and turbidite deposits, are exposed south and north of this melange complex (Fig. 1).

The Pre-Tertiary Luk Ulo melange complex consists of rock units originating from different environments, which are tightly mixed and deformed. 'Exotic' rock blocks, which consist of igneous ultramafic rocks, metamorphic rocks, and sedimentary rocks up to several meters in size, can be mapped as floating fragments in the dark grey-black clay [[21], [22], [23], [24], [20], [19]] This melange complex is unconformably overlain by the Eocene-Oligocene olistostrome deposits, known as Karangsambung Formation (Teok) and Totogan Formation (Tomt) [22,23,[19], [25], [26]].

According to Asikin et al. [19], the difference between these two formations is that the Karangsambung Formation is mostly “clay-shale”, while the Totogan Formation is “clay-breccia/olistostrome”. Stratigraphically, the relationship between the two formations is lateral facies changes from the upper part of the Middle-Late Eocene Karangsambung Formation to the Late Eocene Totogan Formation [27]. These formations generally consist of a mixture of sedimentary fragments and blocks (olistoliths) such as sandstone, siltstone, conglomerate, and Nummulites limestone in a clay matrix [18]. The thickness of the Totogan Formation is about 150 m, and it is thinning to the south [20]. Above the Totogan Formation (Tomt) is the Early-Middle Miocene Waturanda Formation (Tmw), which consists of sandstone and volcanic breccia. This formation is conformably overlain by the Middle Miocene Penosogan Formation (Tmp), which consists of interbedded marl and calcareous sandstone [18,24]. The Penosogan Formation (Tmp) is only observed in the Southern Serayu Mountain [17] and laterally interfingering with the Merawu Series in the Northern Serayu Mountain, comprises the Lutut Members as the lower part and the Rambatan Formation as the upper part (Tmr) [3,25]. The Merawu Series is overlain by the Late Miocene-Pliocene Halang Formation and Kumbang Formation [20,19], also known as the Penyaten Series [3,25]. This formation is distinguished from the Rambatan Formation by the absence of quartz clastic layers and the presence of highly increased andesitic volcanic layers [25].

Lunt (2013) considers the Kumbang Breccia as a proximal facies with a larger grain size, which is laterally equivalent to the finer Halang Formation. The Third Breccia Horizon series unconformably overlies those older Miocene rock layers [20,25]. Then this was known by several different formation names, namely the Peniron Formation (Tpp), which consists of breccia with tufa intercalations; the Kalibiuk Formation (Tpb), which consists of marl, mudstone, and sandy tuff intercalations; and the Tapak Formation (Tpt) comprises calcareous sandstone and marl [20,19].

## 3 Data and methods

A total of fifteen outcrop samples from the Oligocene Totogan Formation have been gathered as potential source rock (Fig. 1, Table 1). The acquired samples showed features typical of fine-grained clastic sedimentary rocks, which were hypothesised to contain organic-rich material. All Totogan Formation claystone samples underwent Total Organic Content (TOC) measurement, as well as the Rock-Eval pyrolysis analysis to examine the S_1_, S_2_, S_3_, and Tmax (°C) in order to calculate the hydrogen index (HI), production yield (PY), and production index (PI) (Table 2). Then, gas chromatography (GC) and gas chromatography-mass spectrometry (GC-MS) analyses were conducted for several selected rock samples (Table 3). However, the GC and GC-MS data of Lawen oil (LW-05A) utilises data published by Praptisih [12].

**Table 1:** Availability data for this research.

**No**	**ID Sample**	**Formation**	**Type**	**TOC - Rock-Evalpyrolysis**	**GasChromatography**	**Liquid** **Chromatography**	**GC-MS** **Saturated**
1	KG-04	Totogan Fm.	Outcrop	✓	✓	–	✓
2	KT-04	Totogan Fm.	Outcrop	✓	–	–	–
3	TTG-01	Totogan Fm.	Outcrop	✓	–	–	–
4	TTG-02	Totogan Fm.	Outcrop	✓	–	–	–
5	G-01	Totogan Fm.	Outcrop	✓	–	–	–
6	G-03	Totogan Fm.	Outcrop	✓	–	–	–
7	G-04	Totogan Fm.	Outcrop	✓	–	–	–
8	G-06	Totogan Fm.	Outcrop	✓	–	–	–
9	CG-02	Totogan Fm.	Outcrop	✓	–	–	✓
10	CG-06A	Totogan Fm.	Outcrop	✓	–	–	✓
11	KB-01	Totogan Fm.	Outcrop	✓	✓	–	✓
12	TB-01	Totogan Fm.	Outcrop	✓	–	–	–
13	ST-01	Totogan Fm.	Outcrop	✓	–	–	–
14	WR-04	Totogan Fm.	Outcrop	✓	–	–	–
15	ST-02	Totogan Fm.	Outcrop	✓	–	–	–
16	Lawen Oil (LW-05A)	–	Oil Seep	–	✓	✓	✓

**Table 2:** TOC and Rock-Eval pyrolysis data of the Totogan Formation in the Banjarnegara and surrounding areas.

No	Sample ID	Formation	TOC (wt%)	S1	S2	S3	PY	PI	Tmax (°C)	HI	OI
				(mg HC/g rock)							
1	KG-04	Totogan Fm.	1.00	0.07	0.86	–	0.93	0.08	439	86	–
2	KT-04	Totogan Fm	1.42	0.06	0.77	–	0.83	0.07	405	54	–
3	TTG-01	Totogan Fm	0.54	0.05	0.07	0.00	0.12	0.42	425	13	–
4	TTG-02	Totogan Fm	0.43	0.01	0.02	0.01	0.03	0.33	420	5	2
5	G-01	Totogan Fm	0.34	0.01	–	–	–	1.00	–		–
6	G-03	Totogan Fm	0.42	0.02	0.06	–	0.08	0.25	426	14	–
7	G-04	Totogan Fm	0.48	0.03	0.00	–	0.03	1.00	–		–
8	G-06	Totogan Fm	0.49	0.03	0.14	–	0.17	0.18	420	29	–
9	CG-02	Totogan Fm	1.00	0.43	0.82	0.15	1.25	0.34	460	82	15
10	CG-06A	Totogan Fm	0.93	0.19	0.15	0.08	0.34	0.56	477	16	9
11	KB-01	Totogan Fm	0.78	0.34	0.29	0.08	0.63	0.54	489	37	10
12	TB-01	Totogan Fm	0.61	0.36	0.25	0.20	0.61	0.59	396	41	33
13	ST-01	Totogan Fm	0.65	0.04	0.38	–	0.42	0.42	472	59	–
14	WR-04	Totogan Fm	0.60	0.03	0.32	–	0.35	0.09	431	53	–
15	ST-02	Totogan Fm	2.12	0.10	1.77	–	1.87	0.05	432	84	–

**Table 3:** Biomarker data from Liquid Chromatography, Gas Chromatography, and Gas Chromatography Analysis of the research area.

		CG-02	CG-06A	KB-01	KG-04	The Lawen oil
		(Rock Extract)	(Rock Extract)	(Rock Extract)	(Rock Extract)	(LW-05A)
Liquid Chromatography	Saturates (%)	–	–	–	–	70.49
	Aromatics (%)	–	–	–	–	22.67
	NSO (%)	–	–	–	–	6.83
	Asphaltenes (%)	–	–	–	–	0.01
	Sat./Aro. Ratio	–	–	–	–	3.11
Gas Chromatography	Pris/Phy	–	–	2.6	3.64	2.66
	Pris/nC_17_	–	–	0.57	1.64	1.24
	Phy/nC_18_	–	–	0.22	0.37	0.53
	nC_31_/nC_19_	–	–	0.2	0.43	0.18
	CPI	–	–	1	1.05	1.06
GC-MS (Terpanes *m*/*z* 191)	C_24_/C_23_ tricyclic terpane	0.73	0.70	0.70	1.23	1.00
	C_21_/C_22_ tricyclic terpane	0.44	0.44	0.36	0.88	0.84
	C_19_/(C_19_+C_23_) tricyclic terpane	0.23	0.22	0.35	0.45	0.56
	C_23_/C_21_ tricyclic terpane	1.24	1.26	0.89	1.00	0.93
	C_29_ norhopane/C_30_ hopane	0.78	1.00	0.74	0.67	0.46
	Oleanane Index (%)	34.50	40.66	23.03	64.23	52.85
	Gammacereane Index (%)	0.00	0.00	0.00	0.00	2.52
	Tm/Ts	0.31	0.17	0.62	0.37	0.39
	C_29_ moretane/C_29_ hopane	0.28	0.14	0.19	0.10	0.23
	C_30_ moretane/C_30_ hopane	0.19	0.11	0.21	0.11	0.14
	C_29_/(C_29_ +C_30_) hopane	43.85	49.89	42.49	40.04	31.73
	22S/(22S + 22R) C_32_ homohopane	0.75	1.32	1.19	3.91	0.43
GC-MS (Sterane *m*/*z* 217)	C_27_ regular strerane (%)	43.03	51.86	38.07	36.76	33.65
	C_28_ regular strerane (%)	33.74	19.42	19.47	12.56	31.36
	C_29_ regular strerane (%)	23.23	28.72	42.46	50.68	35.00
	C_29_/C_27_ sterane	0.54	0.55	1.12	1.38	1.04
	C_27_/C_29_ sterane	1.85	1.81	0.90	0.73	0.96
	C_27_/(C_27_+C_29_) sterane	64.94	64.36	47.28	42.04	49.02
	Total Sterane/Total Hopane	1.99	0.36	0.23	0.25	0.29
	Total Hopane/Total Sterane	0.50	2.78	4.35	4.00	3.45
	Diasterane/Sterane	0.17	0.72	0.35	0.45	0.50
	20S/(20S + 20R) C_29_ sterane	0.05	0.39	0.32	0.25	0.42
Relative Percentages of Major Biomarkers	% Total Hopanes	26.96	28.33	28.95	28.20	28.08
	% Total C_30_ Resins + OL	3.04	3.90	2.30	17.60	11.77
	% Gammacereane	0.00	0.00	0.00	0.00	0.24
	% Total Drimanes + Rearranged	16.46	57.44	62.14	47.27	51.75
	% Total Steranes	45.65	6.02	4.89	4.77	5.45
	% Total Diasteranes	7.90	4.31	1.72	2.15	2.71

The Rock-Eval II Instrument was employed to assess the ability of the potential source rocks to generate petroleum and their level of thermal maturity. The process involves analysing a powdered rock sample, weighing between 90 and 130 mg, by subjecting it to controlled heating within the 300–600 °C temperature range. Prior to heating, any gases present in the sample are eliminated at 90 °C temperature. This assay provides a rapid assessment of the levels of soluble and volatile organic compounds, the quantity of pyrolyzable organic matter, and the degree of thermal maturity. The results are reported in mg/g for the parameters S_1_, S_2_, and S_3_ and in degrees Celsius (°C) for Tmax. The hydrogen index (HI) is determined by dividing the weight of S_2_ by the weight of total organic carbon (TOC) in the sample, measured in mg HC/g TOC. The oxygen index (OI) is determined by the S_3_/TOC (mg CO2/g TOC) value.

Gas chromatography (GC) analysis was performed on the whole oil and extracts obtained from the fractionation process. The instrument used for the analysis was an HP 5890 Series II Gas Chromatography Instrument equipped with a flame ionisation detector (FID). The temperature program ranges from 28 °C to 280 °C, with a heating rate of 6 °C per minute, using the CP-Sil-5CB glass capillary column and hydrogen as the carrier gas.

The biomarkers, also called biological markers, are beneficial in providing insights into various biological processes and confirming the presence of life. This tool is incredibly valuable and effective for conducting source-to-oil or oil-to-oil correlations. The GC-MS analysis was conducted to identify biomarkers in saturated extracts from oil seepage and source rock samples. The GC-MS analysis was performed through a PerkinElmer Clarus 600 GC instrument, which was attached to a Clarus SQ 8C mass spectrometry detector and a DB-5MS column. The data being gathered includes the saturated fraction ion *m*/*z* 191 for terpane and *m*/*z* 217 for sterane. The peak identifications for terpane and sterane can be seen in appendix A and appendix B, respectively.

## 4 Results

### 4.1 TOC and Rock-Eval pyrolysis

Table 2 shows the total organic carbon (TOC) content measurement result and Rock-Eval pyrolysis analysis of 15 claystone samples from the Totogan Formation. The TOC content of those claystones ranges from 0.34 to 2.12 wt%, with an average value of 0.79 wt%. The remaining hydrocarbon potential (S_2_) of 15 rocks varies from 0.00 to 1.77 mg HC/g and an average value of 0.42 mg HC/g rock. The hydrogen index (HI) value is mostly low, with only three samples showing values above 80 mg HC/g TOC. The range of Tmax value for those samples is 396 °C–489 °C, with an average of 437.85 °C.

### 4.2 Liquid chromatography

The Lawen oil (LW-05A) comprises saturate, aromatic, NSO, and asphaltene fractions, accounting for 70.49 %, 22.67 %, 6.83 %, and 0.01 %, respectively. It indicates no signs of biodegradation in the oil seep samples. The proportion between saturate and aromatic fractions is 3.11. Table 3 displays the result of the liquid chromatography analysis.

### 4.3 Gas chromatography (GC)

**Fig. 2:**
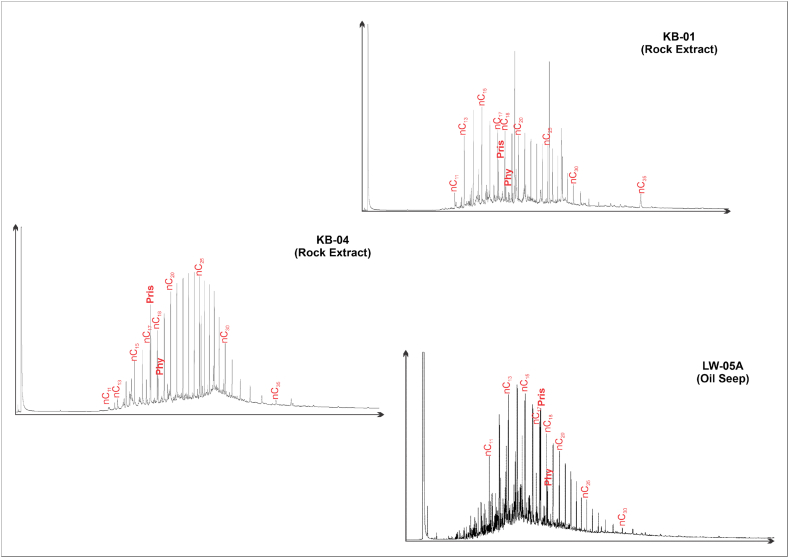
Gas chromatography data of rock extract from Totogan Formation and seepage sample (LW-05A).

GC analysis was conducted on the bitumen extracted from two rock samples (KB-01 and KG-04) and an oil seepage sample (Lawen Oil). The relative abundance of the n-alkanes in each sample from the gas chromatography analysis is shown in Fig. 2. The gas chromatography profile of the bitumen sample is characterised by n-alkanes ranging from nC_11_ to nC_34_. Meanwhile, the Lawen oil exhibits the characteristics of n-alkanes ranging from nC_8_ to nC_35_. Fig. 2 and Table 3 show that the ratio of pristane to phytanes of extracted bitumen and oil seepage sample is > 2, ranging from 2.60 to 3.64.

### 4.4 Gas chromatography-mass spectrometry (GC-MS)

GC-MS analysis was performed on bitumen extracted from four samples: CG-02, CG-06A, KB-01, and KG-04, which were collected from the Totogan Formation. Additionally, one sample of oil seepage (Lawen Oil) that was analysed by Praptisih [12].

#### 4.4.1 Mass fragmentogram of terpane biomarkers (m/z 191)

**Fig. 3:**
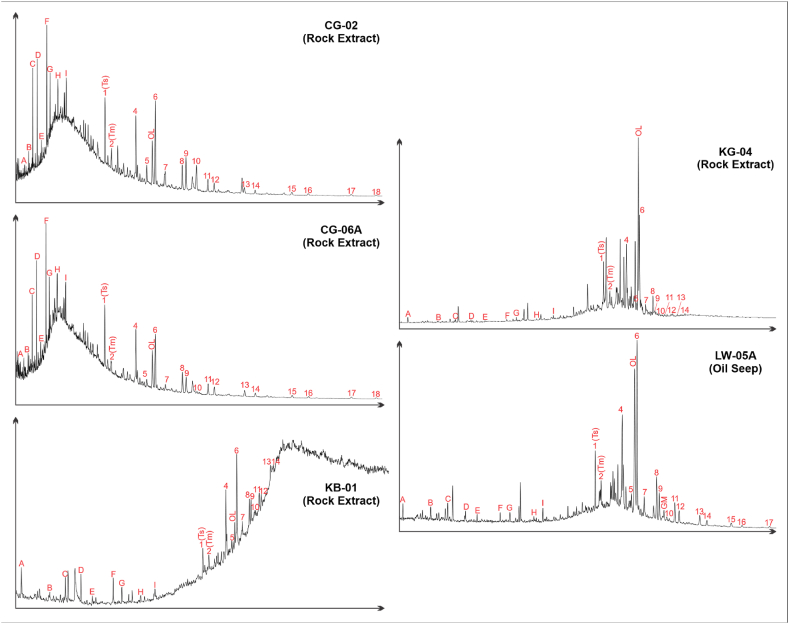
Extended mass fragmentogram of terpanes biomarkers (*m*/*z* 191). Peak identification of terpanes is shown in Appendix A.

The *m*/*z* 191 fragmentogram of saturated hydrocarbon shows that the bitumen from rock extract CG-02, CG-06A, KB-01, KG-04, and Lawen Oil (LW-05A) exhibits a distribution of tricyclic terpanes with carbon atoms that vary from C_19_ to C_26_ (peak A-I) (Fig. 3). The abundance of tricyclic terpanes of bitumen CG-02, CG-06A, and KB-01 is relatively higher than its pentacyclic terpane compound, while the bitumen KG-04 and oil seepage LW-05A showed the opposite (Fig. 3). Aside from that, the mass fragmentogram of bitumen CG-02 and CG-06A also show a high abundance of C_23_ (peak F) relative to C_19_ and C_20_ tricyclic compounds (peak B and C, respectively), while the bitumen KB-01, KG-04, and oil sample LW-05 exhibit higher abundance of the C_19_ and C_20_ (B and C, respectively) relative to C_23_ tricyclic compound (peak F).

Almost the bitumen and oil seepage samples show the predominance of C_30_ hopane (peak 6) relative to C_29_ norhopane (peak 4), except for CG-06A, which have different characteristics, namely peak of C_29_ norhopane and C_30_ hopane are relatively the same high. Hence, the ratio between C_29_ norhopane and C_30_ hopane is 1 (Fig. 3 and Table 3). The trisnorneohopane (Ts) is higher than trisnorhopane (Tm) in all samples. Consequently, the Tm/Ts ratio is lower than 1, ranging from 0.17 to 0.62 (Fig. 3 and Table 3). The C_29_ and C_30_ moretanes (peaks 5 and 7) are extremely low relative to C_29_ and C_30_ hopanes (peaks 4 and 6), respectively (Fig. 3), with ranges of 0.10–0.28 and 0.11–0.21 (Table 3). The abundance of the Oleanane compound for the extracted bitumen and oil seepage is present in significant amounts that are reflected by peak OL on mass fragmentogram *m*/*z* 191 (Fig. 3), with an Oleanane Index range between 23.03 % and 64.23 % (Table 3). Gammacerane (peak GM) compounds are absent in all extracted bitumen samples, and there are low amounts in oil seepage LW-05A (Table 3). The C_31_ to C_35_ homohopanes (22S and 22R) compound was observed completely in bitumen CG-02 and CG-06 (peaks 8 to 18 in Fig. 3). Meanwhile, in the oil seepage, LW-05A and the remaining extracted bitumen sample (KB-01 and KG-04) were only observed to peak at 17 (C_35_ homohopane 22S) and to peak 14 (C_33_ homohopane 22R), respectively. Only samples CG-02, CG-06, and LW-05A show regular stair-step progression patterns of C_31_ to C_31+_, while the other samples exhibit irregular patterns (Fig. 3). The C_31_ to C_31+_ 22S homohopanes are high relative to their 22R homohopanes on almost every sample, except for sample CG-02 (especially for C_31,_ which shows the 22R homohopanes are high relative to its 22S) and sample KG-04 (that show relatively little response in C_32+_).

#### 4.4.2 Mass fragmentogram of Sterana Biomarkers (m/z 217)

**Fig. 4:**
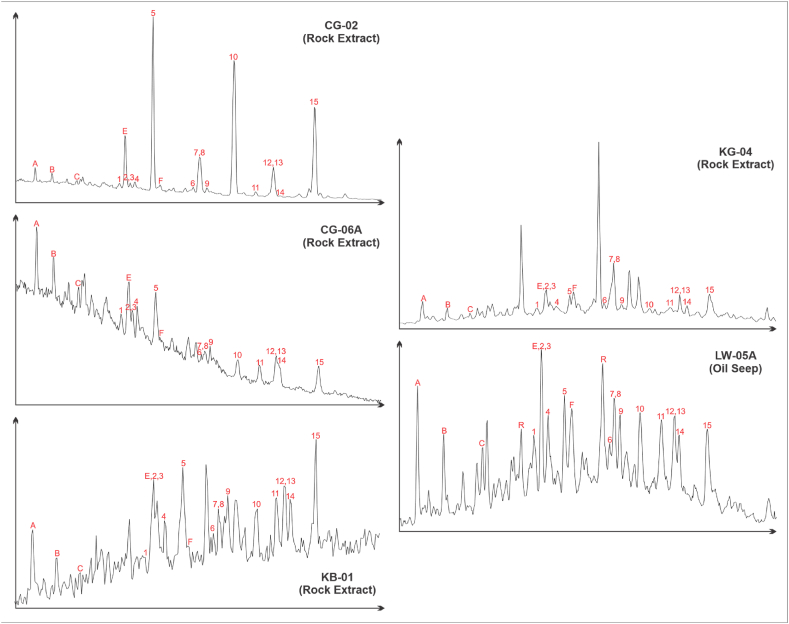
Partial Mass Fragmentogram of Sterana Biomarkers (*m*/*z* 217). Peak identification of terpanes is shown in Appendix B.

The *m*/*z* 217 fragmentogram of saturated hydrocarbon (Fig. 4) shows that steranes (Peak 1–15) are more abundant than diasteranes (Peak A – E), which is reflected by the diasteranes/steranes ratio lower than 1 with a range of 0.17–0.72 (Table 3). Regarding on the regular sterane composition (C_27_-C_28_-C_29_) (Table 3), the bitumen sample of CG-02 and CG-06 exhibit that C_27_ sterane is more abundant than their C_29_ sterane, with a range of 43.03 %–51.86 % and 23.23 %–28.72 %, respectively. Furthermore, the C_29_ regular sterane of KB-01, KG-04, and Lawen Oil Seepage (LW-05A) is more predominant than their C_27_ regular sterane (Table 3). It is also reflected by the ratio of C_27_/C_29_ regular sterane. Most of the sample has a hopane/sterane ratio value higher than 1, except for the bitumen sample of CG-06A, which is 0.5.

## 5 Discussion

### 5.1 Biodegradation of oil seepage and bitumen extract

The biodegradation of oil seeps and bitumen extract samples was analysed through the distribution of biomarker compounds, including n-alkanes, isoprenoids, hopanes, and steranes. The biodegradation scales were derived from qualitative evaluations of biodegraded oils and bitumen extract, emphasising the differential loss of compounds across various biodegradation ranks [[28], [29], [30], [31]].

The CG-02 and CG-06A bitumen extract samples exhibit a complete absence of *n*-alkanes and isoprenoids. As a result, the Pr/Ph, Pr/C_17_, and Ph/C_18_ ratios were unable to be calculated. The samples exhibited no signs of sterane biodegradation, as the C_27_ sterane appeared to be preserved. The distribution of hopane compounds in these samples did not show any characteristics of bacterial alteration, as the 17α homohopane compounds were found in complete ranges up to C_35_. These compounds exhibit significant resistance to biodegradation, even at elevated levels [28]. Therefore, when the *n*-alkanes and isoprenoids from the extract are totally absent and the hopane and sterane remain intact, a certain extent of biodegradation is indicated in PM 5 [29,31].

The Lawen oil seep and KG-04 bitumen extract biodegraded slightly. The *n*-alkanes and isoprenoids were well preserved in these samples. High-numbered *n*-alkanes dominated the samples, while low-numbered ones were nearly absent. A slightly unresolved complex mixture (UCM) was also present. In the oil seep and bitumen extract samples, nC_1_-nC_10_ is removed, whereas nC_11_-nC_14_ remains low. The dominance of pristane over nC_17_ molecules (ratio of Pr/nC_17_ > 1) and the distribution pattern of n-alkanes in these samples suggest biodegradation slightly occurred. This circumstance happened because the partially removed nC_17_ molecule was attacked faster than the pristane [28]. The extract from KB-01 bitumen demonstrated preferable preservation of *n*-alkanes and isoprenoids, as evidenced by the Pr/nC_17_ and Ph/nC_18_ ratios remaining below 1. However, nC_1_ to nC_10_ were nearly nonexistent. Sterane and hopane biomarkers for all of these samples were well preserved, suggesting that Lawen oil seep, KG-04, and KB-01 bitumen extracts samples underwent PM 1–2 scale of biodegradation [29,31].

### 5.2 Geochemical characteristics of Totogan Fm. as potential source rock

#### 5.2.1 Organic matter richness and generative potential of source rock

**Fig. 5:**
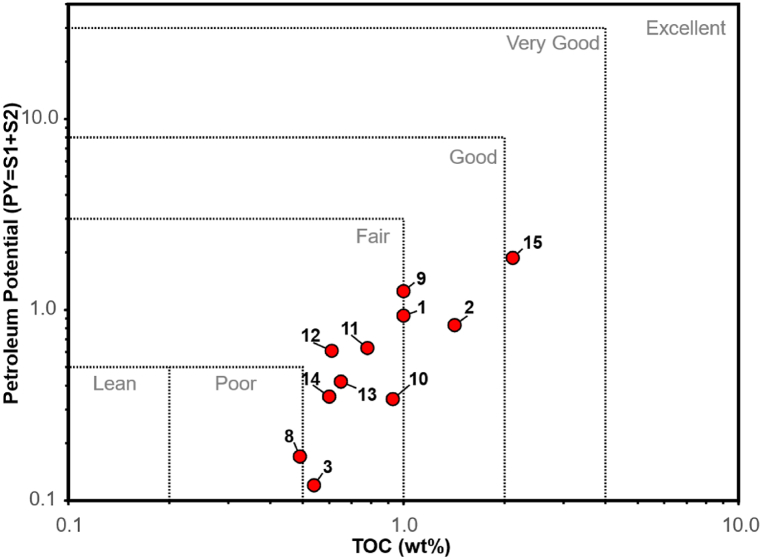
Cross-plot of total generation potential yield (PY) versus total organic carbon (TOC) shows that the Totogan Formation has poor to good generative potential as organic source rocks (after Al-Matary et al. [33], and Hakimi et al. [34]).

The organic matter richness and source rock generative potential of Totogan Formation, as a potential source rock, was evaluated using the TOC and Rock-Eval pyrolysis data (Table 2). The TOC value range of 15 samples shows that the organic matter richness of this formation is classified as fair to good petroleum potential [32]. Furthermore, the cross-plot of total generation potential yield (PY) against total organic carbon (TOC) [33,34] shows that the Totogan Formation has poor to good generative potential as organic source rocks (Fig. 5).

#### 5.2.2 Kerogen type of source rock

**Fig. 6:**
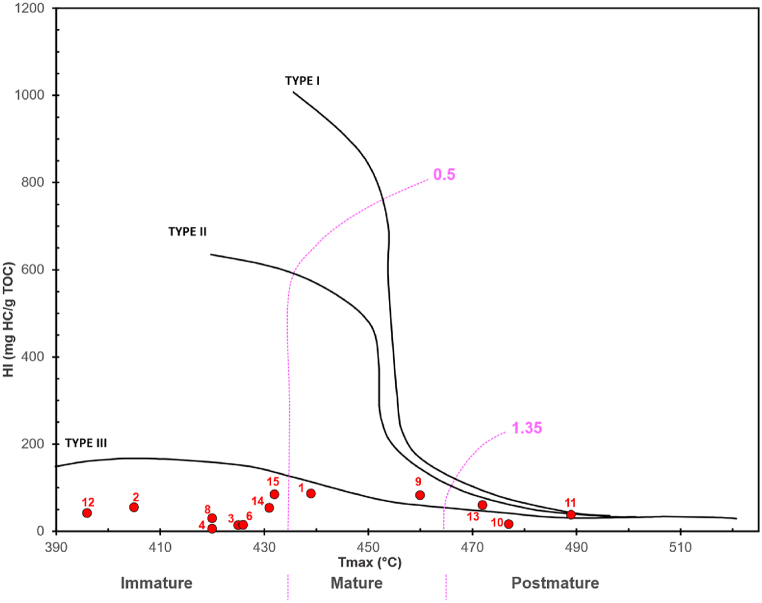
A cross plot of hydrogen index (HI) versus Tmax for the Totogan Formation shows that the Totogan Formation is composed of Type III kerogen with thermal maturity stages varying from immature to post mature (Espitalie et al. [35] and Mukhopadhyay et al. [36]).

Based on the results of Rock-Eval pyrolysis, GC, and GC-MC analyses, it can be inferred that the sample of the Totogan Formation exhibits different kerogen types, sources, depositional environment, and maturity of organic matter. Based on Rock-Eval pyrolysis data, depicted by the hydrogen index (HI) versus Tmax diagram (Fig. 6), the WR04, ST02, KT04, TB05, TTG01, TTG02, G03, G06 samples were then grouped as type III kerogen with the 396–432 °C Tmax value, indicating a thermally immature level. The KG-04, CG-02, and CG-06A have a HI value of 16–86 mg HC/TOC and Tmax value of 439 °C–477 °C, indicating this sample is classified as type III to type IV kerogen with the level of mature thermally. The other three samples, ST01, CG06A, and KB-01, have HI values of 16–59 mg HC/TOC (kerogen type III) and the Tmax value range of 472–489 °C, categorised as post-mature [32].

The hydrogen index value from pyrolysis data (HI lower than 100) shows that the kerogen type of all Totogan Formation claystone samples is type III (gas-prone) to IV (inert) [32]. However, a low hydrogen index value can also be due to a portion of the sedimentary organic matter converted to hydrocarbons when experiencing a higher level of maturity [37]. In addition, for source rocks with mixed sources of organic matter like the Totogan Formation, it is not reliable to determine the kerogen type based solely on the hydrogen and oxygen indexes [38]. Furthermore, other factors that cause low HI values include the outcrop's weathering conditions, the composition of the kerogen types, and the pyrolytic adsorption effects attributed to the clay matrix [30,[39], [40], [41], [42]]. The analysis performed on the Cikalong Formation, which is equivalent in age to the Totogan Formation, indicates this condition [30]. This formation, derived from rock evaluation results, indicates Type IV kerogen because of a low HI value. However, the visual kerogen type study indicates a relative percentage of maceral composition consistent with Type II/III kerogen. Therefore, it is assumed that the Totogan Formation is classified as type II/III kerogen, which tends to generate a mixing of gas and oil, rather than type III to type IV kerogen.

#### 5.2.3 Organic matter source and depositional environment of source rock

**Fig. 7:**
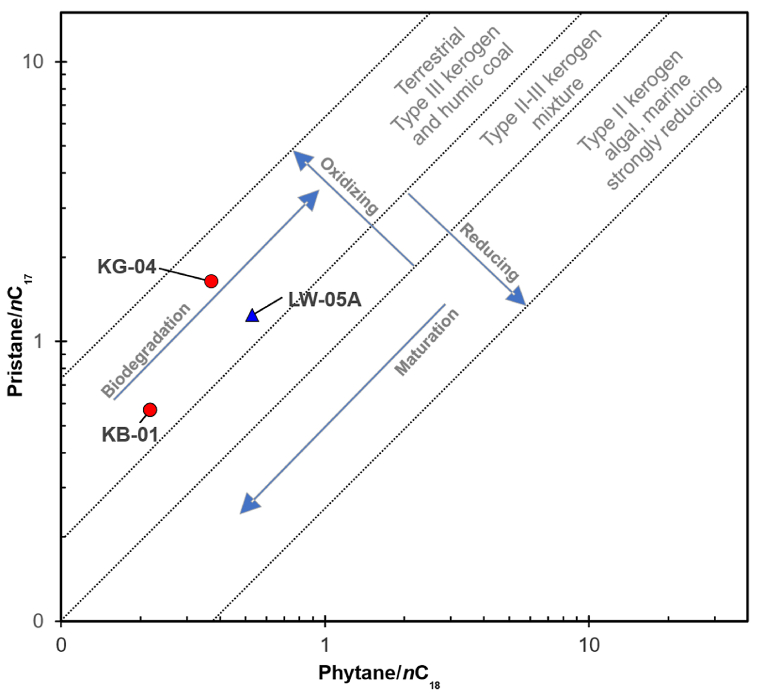
A plot of pristane/nC_17_ versus phytane/nC_18_ (after Shanmugam [49]).

Biomarker fingerprint n-alkanes from GC data (Fig. 2) reveals that the potential source rock (KB-01 and KG-04) shows a relatively high concentration of higher molecular weight components (nC_22+_) that indicate input from higher plant organic debris [[43], [44], [45], [46]]. The higher value of the pristane/phytane ratio (Table 3), namely 2.6–3.64, indicates that the source rock was deposited under suboxic to oxic conditions [47,48]. The plot of pristane/nC_17_ versus phytane/nC_18_ [49] suggests that the extracted bitumen produced from two extracted bitumen (KB-01 and KG-04) derived mainly from terrestrial type III kerogen and humic coal (Fig. 7).

**Fig. 8:**
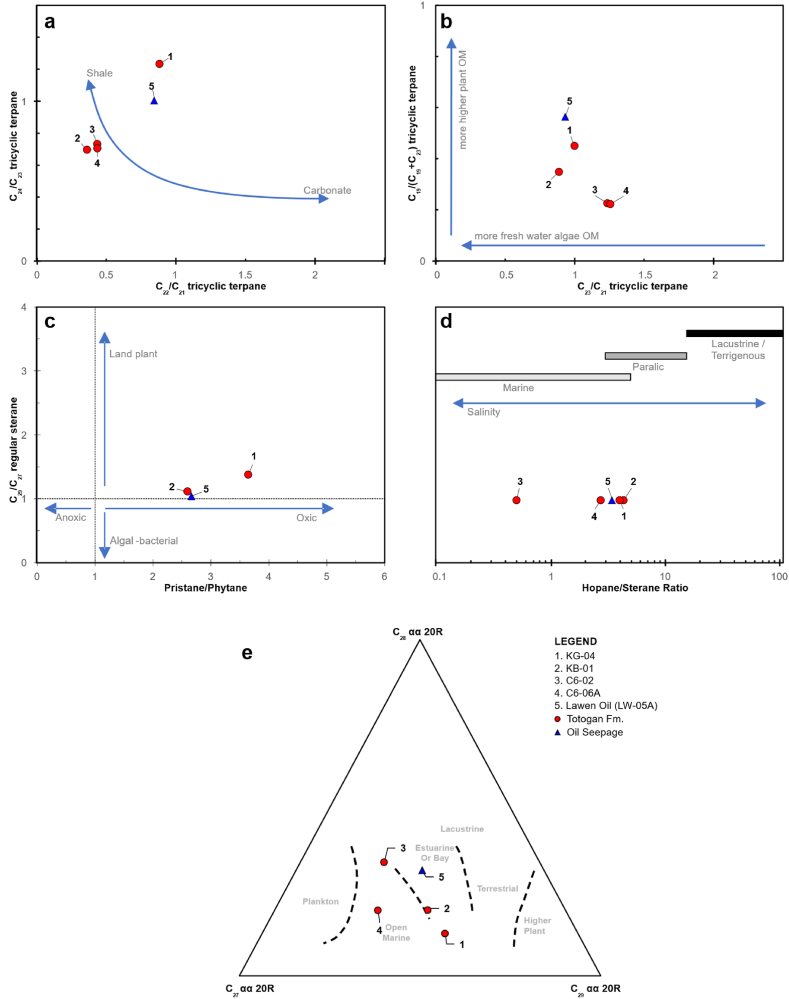
a) Cross-plot of C_24_/C_23_ versus C_22_/C_21_ tricyclic terpane ratio (after Peters et al. [48]), b) Cross-plot of C_19_/(C_19_+C_23_) versus C_23_/C_21_ tricyclic terpane ratio, c) Cross-plot of C_29_/C_27_ regular steranes against Pr/Ph (after Adegoke et al. [51], d) Correlation of source facies with the total hopane/sterane ratio (after Hsu et al. [52]), e) C_27_-C_28_-C_29_ regular steranes ternary diagram of Totogan Formation (after Huang and Meinschein [53].

The compounds of C_23_ (Peak F) tricyclic terpane are comparatively more significant than C_19_ and C_20_ tricyclic terpane (Peak B and C), following a C_20_<C_21_<C_23_ pattern (Fig. 3). This pattern suggests that the source facies of CG-02 and CG-06A, derived from marine algae OM [46,50]. It differs from KB-01 and KG-04, which indicates they may have derived mainly from terrestrial high plant OM [46,50]. It is supported by the cross plot of C_19_/(C_19_+C_23_) tricyclic terpane against the C_23_/C_21_ tricyclic terpane ratio (Fig. 8b).

Furthermore, the occurrence of 18α(H)-Oleanana and C_30_ resin bicadinanes compounds was observed in rock extract samples, indicating the existence of resin material originating from terrestrial sediment sources, mainly flowering plants that emerged since the Cretaceous period [48,54] Table 3 shows that the Oleanana Index (Oleanane/(Oleanane + C_30_ Hopane)) is greater than 20 %. This indicates that all of the bitumen extracts came from tertiary rock [48,[54], [55], [56], [57]]. The low Gammacerane Index values (gammacerane/(gammacerane + C_30_ hopane)) suggest the absence of hypersaline conditions during deposition [[58], [59], [60]].

The mass fragmentogram of biomarker sterane (*m*/*z* 217) from samples CG-02 and CG-06A (Fig. 4 and Table 3) show a normal distribution showing that C_27_ sterana (43.03–51.86 %) have a greater than C_29_ sterane (23.23–28.72 %) indicate the algae marine is the primary source for the organic material [[53], [61], [62], [63]]. On the other hand, the sample of KB-01 and KG-04 have a greater proportion of C_29_ sterane (42.46–50.68 %) than C_27_ sterana (36.76–38.07 %) that indicates this sample mainly derived from high plant input [[53], [61], [62], [63]]. The low steranes/hopanes of KB-01 and KG-04 samples (<1) are also indicative of terrigenous of high plant organic matter input [63,64]. In addition, the ratio of hopane/sterane can be used to determine source facies [52]. The hopane/sterane ratio of CG-02 and CG-06A samples implies marine source facies, whereas KB-01 and KG-04 imply paralic source facies (Fig. 8d). Based on the percentage of the total amount of carbon and plotting it into a triangular diagram [53] (Fig. 8e), it can be inferred that CG-02 and CG-06A samples were deposited in an open marine environment with input mainly from algae organic matter. In contrast, the KB-01 and KG-04 are estuarine or bay deposits with input mainly from high plant organic matter. This interpretation is also supported by the cross-plot of the ratio of pristane/phytane against the ratio of C_29_/C_27_ regular steranes [51] (Fig. 8c).

#### 5.2.4 Maturity of source rock

**Fig. 9:**
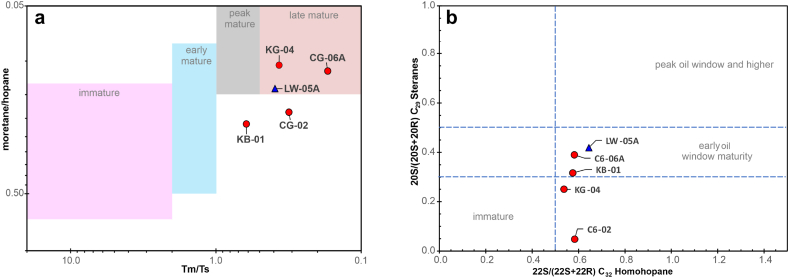
a) Cross-plot of Tm/Ts against moretane/hopane (after Miles [65]) and b) cross-plot of 22S/(22S + 22R) C_32_ homohopane versus 20S/(20S + 20R) C_29_ sterane [66] that the maturity level of the oil seepage and the bitumen extract from the source rock samples.

The thermal maturity of the Totogan Formation was examined based on the Rock-Eval pyrolysis data and biomarker data (GC and GC-MS). According to the range of Tmax data (Table 2), the Totogan Formation is immature to post-mature thermally (396 °C–489 °C) [32]. The CPI values of the extracted bitumen from Totogan Formation claystone represented by KB-01 and KG-04 samples were between 1.00 and 1.05 (Table 3). Based on studies by Bray & Evans in 1961, the CPI values are close to 1, indicating that all the samples are mature thermally [48]. It is also supported by the cross plot of Tm/Ts against mortepane/hopane [65], which shows that samples CG-02, CG-06A, KB-01, and KG-01 fall in zones peak mature to late mature (Fig. 9).

The Totogan Formation has a wide range of thermal maturity levels, as shown by the Tmax value (immature to post-mature). This condition can be due to limited sample load, clay content in the samples, weathering or oxidation, over-mature conditions, carryover effects, and high concentrations of hydrogen, sulfur, and uranium in the samples, which can result in lower or higher Tmax values than expected [67,68]. This condition was observed in sample KB-01, which showed a Tmax value of 489 °C, indicating post-maturity, at which temperature the biomarker should have been destroyed. Nonetheless, the biomarker remains detectable. So, the Tmax value is unreliable for determining the source rock's maturity level. The increased Tmax anomaly value could be attributable to the characteristics of the clay-rich sample and is additionally affected by weathering and oxidation processes [[67], [68], [69]].

### 5.3 Geochemical characteristics of oil seepage

The analysis of the Lawen oil seepage revealed an absence of biodegradation indicators in the oil samples. The oil seepage samples exhibited a significant saturated fraction exceeding 70 %, suggesting the presence of paraffin-naphthenic oil, potentially resulting from thermal alteration indicative of high maturity. Subsequently, the saturated/aro ratio suggested a potential origin from marine shale [46].

The ratio of pristane compared to phytane that is greater than 1 indicates the Lawen Oil derived from source rock deposited under suboxic to oxic conditions [47,48]. The plot of pristane/nC_17_ versus phytane/nC_18_ expresses that the oil seepage sample (LW-05A) derived from terrestrial type III kerogen and humic coal (Fig. 7). Furthermore, the cross-plot of C_24_/C_23_ tricyclic terpane ratio versus C_22_/C_21_ tricyclic terpane ratio [48] indicate that the lithology of source rock is shale (Fig. 8a). From calculating the percentage of the total amount of carbon and plotting it into a triangular diagram [53] (Fig. 8e), demonstrate that the source rock of the Lawen Oil was deposited in estuarine or bay deposit with input mainly from high plant organic matter. It is supported by the hopane/sterane ratio value that indicates the source is paralic facies (Fig. 8d). The low steranes/hopanes of this samples (<1) are indications of terrestrial high plant organic matter input [63,64]. Thus, it can be concluded that the Lawen Oil is derived from source rock mainly composed of terrestrial type III kerogen, with the high plant organic matter as the source, and was deposited in an estuary or bay environment under suboxic to oxic conditions.

In addition, the Oleanana Index of this oil seepage sample that is greater than 20 %, indicates the Lawen Oil is generated from Cretaceous or younger source rock with higher plant angiosperm marker [48,[54], [55], [56], [57]]. The low Gammacerane index indicates that the source rock as the source of this oil seepage was not deposited under hypersaline conditions [[58], [59], [60]]. Furthermore, this oil seepage LW-05A was generated from a source rock, which thermally matured based on the value of CPI (CPI = 1.06) [70], the cross plot of Tm/Ts against mortepane/hopane [65] and 22S/(22S + 22R) C_32_ homohopane versus 20S/(20S + 20R) C_29_ sterane [66] (Fig. 9).

### 5.4 Oil – source rock correlation

The oil–source rock correlation between samples from the Totogan Formation and the Lawen Oil is conducted by comparing the kerogen type, depositional environment, sources of organic material, and their maturity level [32,41,46,48,61,71,72]. According to the analysis of available biomarker data, the Totogan Formation claystone can be categorised into two distinct types. The first claystone type was deposited in estuary or bay environments under suboxic to oxic conditions and composed of type III kerogen with organic material originating from higher plant material. The second type is claystone, which was deposited in an open marine environment in suboxic to oxic conditions and received input mainly from organic algal material. Therefore, it can be concluded that the oil seepage of Lawen Oil is generated from the first claystone type of Totogan Formation that was deposited in an estuarine or bay environment with suboxic to oxic conditions and the organic matter derived from high plant material (KB-01 and KG-04). It is supported by the cross-plot of pristane/nC_17_ versus phytane/nC_18_ (Fig. 7), the cross-plot of tricyclic terpane C_24_/C_23_ versus C_22_/C_21_ ratio (Fig. 8a), the cross-plot of C_19_/(C_19_+C_23_) versus C_23_/C_21_ tricyclic terpane ratio (Fig. 8b)), the cross-plot of C_29_/C_27_ regular steranes against Pr/Ph (Fig. 8c), the correlation of source facies with the total hopane/sterane ratio (Fig. 8d), the ternary diagram of Huang and Meinschein (Fig. 8e), and the cross-plot of terpane maturity parameter (Fig. 9). Then, the Oleanane Index and the Gammacerane Index values are nearly identical between the suspected source rock (KG-04) and Lawen Oil samples. Namely, the Oleanane Index is more than 20 %, while the Gammacerane Index is equally low.

**Fig. 10:**
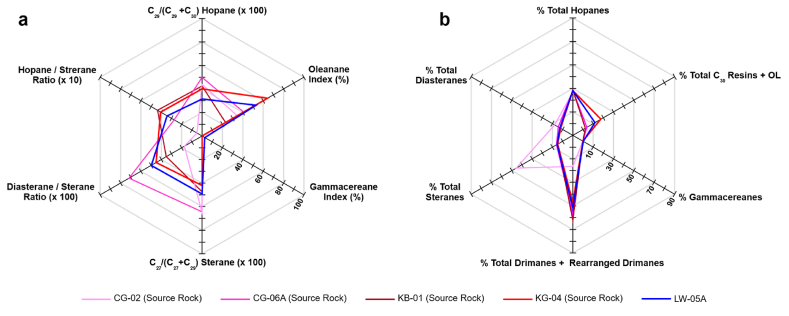
The star diagram shows the distribution of source-related biomarkers (a) and the distribution of major biomarker classes (b) from the source rock and oil seepage sample.

The relationship between the potential source rock of the Totogan Formation with the Lawen Oil was also examined using the star diagram (Fig. 10). The star diagram is a multivariate plot for several parameters from selected biomarker data that was used for oil-to-source rock and oil-to-oil correlation in several publications (i.e., Atwah et al. [73] and Belhaj Mohamed et al. [74]). The chosen parameter that was plotted in the star diagram (Fig. 10a) is a source-related biomarker that is not significantly affected by migration, biodegradation, or thermal maturation [48,75]. The star diagram in Fig. 10a shows that only the bitumen from the rock extract of the KG-04 sample shows a positive correlation with the Lawen Oil. It is supported by the similarity pattern of the mass fragmentogram of terpane *m*/*z* 191 (Fig. 3). The star diagram in Fig. 10b also depicts the positive correlation between KG-04 and the Lawen Oil based on the relative percentage composition of major biomarkers in each potential source rock and oil seepage samples.

This finding refutes the results of previous research by Subroto et al. [8]. Based on biomarker data, Subroto et al. [8] reported that only the Late Miocene-Pliocene Halang Formation positively correlated with the Lawen Oil. However, regarding maturity level, this formation has not yet reached the hydrocarbon generation window. It also accords with previous maturity modelling [9], which showed that only the Oligo-Miocene and Eocene sediments appear to be mature enough to generate hydrocarbons. Thus, in the end, Subroto et al. [8] concluded that the source of this oil seepage LW-05A was the Eocene Wungkal Formation because, from many geochemical aspects, this formation has characteristics similar to the Halang Formation. Unfortunately, this Eocene Wungkal Formation is located in the Bayat area, which is more than 100 km east of the oil seepage location. Therefore, the only probable rock source in this research area is the Totogan Formation.

## 6 Conclusions

The study of the geochemical characteristics of oil seepages and source rock from the Totogan Formation revealed the following conclusion.

• The Totogan Formation is considered to have poor to good petroleum generation potential. It is proven by its total organic carbon content (TOC) of up to 2.21 wt%.
• The Totogan formation claystone is comprised of type II/III kerogen.
• Biomarker data classifies Totogan Formation into two categories. The first is claystone deposited under suboxic to oxic estuary or bay settings with mainly high plant material organic content. The second type was open marine deposit with input mainly from algae organic matter.
• The biomarker composition of the LW-05A oil seepage shows that the source rock mainly consists of high plant organic matter deposited in an estuarine or bay environment under suboxic to oxic conditions.
• The oil seepage of LW-05A has a positive oil-to-source rock correlation with the Totogan Formation based on kerogen type, depositional environment, sources of organic material, and their maturity level. This positive correlation is also supported by multivariate plots of several parameters selected from biomarker data.

The new understanding from the research should help improve knowledge about the petroleum system and encourage further hydrocarbon exploration activities in Central Java and its surroundings. Based on new biomarker data, the Totogan formation, previously not considered a potential source rock in this area, apparently shows a positive correlation with Lawen oil seeps. However, this is only a preliminary study result. Further research is needed to overcome the limitations of this study, such as conducting visual kerogen typing analysis to determine the type of kerogen from the samples. In addition, a systematic geochemical investigation is also needed on numerous Totogan Formation rock specimens to generate a more comprehensive conclusion.

## CRediT authorship contribution statement

**Praptisih:** Writing – review & editing, Writing – original draft, Methodology, Investigation, Conceptualization, Writing – review & editing, Writing – original draft, Methodology, Investigation, Conceptualization. **Yoga Andriana Sendjaja:** Supervision, Methodology, Conceptualization. **Vijaya Isnaniawardhani:** Supervision, Methodology, Conceptualization. **Anggoro Tri Mursito:** Supervision, Methodology, Conceptualization. **Taufik Ramli:** Writing – review & editing, Writing – original draft, Visualization, Methodology, Conceptualization. **Muhammad Maruf Mukti:** Writing – review & editing, Writing – original draft, Supervision. **Rakhmat Fakhruddin:** Writing – review & editing, Writing – original draft, Supervision.

## Data and code availability

Data will be made available on request.

## Declaration of competing interest

The authors declare that they have no known competing financial interests or personal relationships that could have appeared to influence the work reported in this paper.

## Figures and Tables

**Table:** A. Terpane Peak Identification

Peak No.	Carbon No.	Identification
A	19	Tricyclic terpane
B	19	Tricyclic terpane
C	20	Tricyclic terpane
D	21	Tricyclic terpane
E	22	Tricyclic terpane
F	23	Tricyclic terpane
G	24	Tricyclic terpane
H	25	Tricyclic terpane
I	26	Tricyclic terpane
1	27	18α(H),21β(H)-22,29,30-trisnorhopane (Ts)
2	27	17α(H),21β(H)-22,29,30-trisnorhopane (Tm)
3	28	17α(H),21β(H)-28,30-bisnorhopane
4	29	17α(H),21β(H)-30-norhopane
5	29	17α(H),21α(H)-30-normoretane
6	30	17α(H),21β(H)-hopane
7	30	17α(H),21β(H)-moretane
8	31	17α(H),21β(H)-30-homohopane (22S)
9	31	17α(H),21β(H)-30-homohopane (22R)
10	31	17α(H),21α(H)-homomoretane
11	32	17α(H),21β(H)-30,31-bishomohopane (22S)
12	32	17α(H),21β(H)-30,31-bishomohopane (22R)
13	33	17α(H),21β(H)-30,31,32-trishomohopane (22S)
14	33	17α(H),21β(H)-30,31,32-trishomohopane (22R)
15	34	17α(H),21β(H)-30,31,32,33-tetrahomohopane (22S)
16	34	17α(H),21β(H)-30,31,32,33-tetrahomohopane (22R)
17	35	17α(H),21β(H)-30,31,32,33,34-pentahomohopane (22S)
18	35	17α(H),21β(H)-30,31,32,33,34-pentahomohopane (22R)
OL	30	18α(H)-Oleanane
GM	30	Gammacerane

**Table:** B. Sterane Peak Identification

Peak No.	Carbon No.	Identification
A	27	13β(H),17α(H)-Diacholestane (20S)
B	27	13β(H),17α(H)-Diacholestane (20R)
C	28	24-Methyl-13β(H),17α(H)-Diacholestane (20S)
D	28	24-Methyl-13β(H),17α(H)-Diacholestane (20R)
E	29	24-Ethyl-13β(H),17α(H)-Diacholestane (20S)
F	29	24-Ethyl-13β(H),17α(H)-Diacholestane (20R)
1	27	5α(H),14α(H),17α(H)-Cholestane (20S)
2	27	5β(H),14α(H),17α(H)-Cholestane (20R)
3	27	5α(H),14β(H),17β(H)-Cholestane (20R)
4	27	5α(H),14β(H),17β(H)-Cholestane (20S)
5	27	5α(H),14α(H),17α(H)-Cholestane (20R)
6	28	24-Methyl-5α(H),14α(H),17α(H)-Cholestane (20S)
7	28	24-Methyl-5β(H),14α(H),17α(H)-Cholestane (20R)
8	28	24-Methyl-5α(H),14β(H),17β(H)-Cholestane (20R)
9	28	24-Methyl-5α(H),14β(H),17β(H)-Cholestane (20S)
10	28	24-Methyl-5α(H),14α(H),17α(H)-Cholestane (20R)
11	29	24-Ethyl-5α(H),14α(H),17α(H)-Cholestane (20S)
12	29	24-Ethyl-5β(H),14α(H),17α(H)-Cholestane (20R)
13	29	24-Ethyl-5α(H),14β(H),17β(H)-Cholestane (20R)
14	29	24-Ethyl-5α(H),14β(H),17β(H)-Cholestane (20S)
15	29	24-Ethyl-5α(H),14α(H),17α(H)-Cholestane (20R)
R		Cyclic Alkane
